# Unravelling the Phytochemical Composition and the Pharmacological Properties of an Optimized Extract from the Fruit from *Prunus mahaleb* L.: From Traditional Liqueur Market to the Pharmacy Shelf

**DOI:** 10.3390/molecules26154422

**Published:** 2021-07-22

**Authors:** Giustino Orlando, Annalisa Chiavaroli, Sabrina Adorisio, Domenico V. Delfino, Luigi Brunetti, Lucia Recinella, Sheila Leone, Gokhan Zengin, Alessandra Acquaviva, Paola Angelini, Giancarlo Angeles Flores, Roberto Venanzoni, Simonetta Cristina Di Simone, Francesca Di Corpo, Andrei Mocan, Luigi Menghini, Claudio Ferrante

**Affiliations:** 1 Department of Pharmacy, Botanic Garden “Giardino dei Semplici”, Università degli Studi “Gabriele d’Annunzio”, Via dei Vestini 31, 66100 Chieti, Italy; giustino.orlando@unich.it (G.O.); annalisa.chiavaroli@unich.it (A.C.); luigi.brunetti@unich.it (L.B.); lucia.recinella@unich.it (L.R.); sheila.leone@unich.it (S.L.); alessandra.acquaviva@unich.it (A.A.); disimonesimonetta@gmail.com (S.C.D.S.); francesca.dicorpo@studenti.unich.it (F.D.C.); claudio.ferrante@unich.it (C.F.); 2 Section of Pharmacology, Department of Medicine and Surgery, Foligno Nursing School, University of Perugia, 06100 Perugia, Italy; adorisiosabrina@libero.it (S.A.); domenico.delfino@unipg.it (D.V.D.); 3 Department of Biology, Science Faculty, Selcuk University, Campus, Konya 42130, Turkey; 4 Veridia Italia Srl, Via Raiale 285, 65100 Pescara, Italy; 5 Department of Chemistry, Biology and Biotechnology, University of Perugia, 06100 Perugia, Italy; paola.angelini@unipg.it (P.A.); giancarlo.angelesflores@studenti.unipg.it (G.A.F.); roberto.venanzoni@unipg.it (R.V.); 6 Faculty of Pharmacy, “Iuliu HaŢieganu” University of Medicine and Pharmacy, 8 Victor Babeş Street, 400012 Cluj-Napoca, Romania; 7 Laboratory of Chromatography, Institute of Advanced Horticulture Research of Transylvania, University of Agricultural Sciences and Veterinary Medicine, 400372 Cluj-Napoca, Romania

**Keywords:** *Prunus mahaleb* L., phenolic profile, chicoric acid, protective effects, anti-bacterial effects, anti-COVID-19 effects

## Abstract

*Prunus mahaleb* L. fruit has long been used in the production of traditional liqueurs. The fruit also displayed scavenging and reducing activity, in vitro. The present study focused on unravelling peripheral and central protective effects, antimicrobial but also anti-COVID-19 properties exerted by the water extract of *P. mahaleb.* Anti-inflammatory effects were studied in isolated mouse colons exposed to lipopolysaccharide. Neuroprotection, measured as a blunting effect on hydrogen-peroxide-induced dopamine turnover, was investigated in hypothalamic HypoE22 cells. Antimicrobial effects were tested against different Gram+ and Gram- bacterial strains. Whereas anti-COVID-19 activity was studied in lung adenocarcinoma H1299 cells, where the gene expression of ACE2 and TMPRSS2 was measured after extract treatment. The bacteriostatic effects induced on Gram+ and Gram- strains, together with the inhibition of COX-2, TNFα, HIF1α, and VEGFA in the colon, suggest the potential of *P. mahaleb* water extract in contrasting the clinical symptoms related to ulcerative colitis. The inhibition of the hydrogen peroxide-induced DOPAC/DA ratio indicates promising neuroprotective effects. Finally, the downregulation of the gene expression of ACE2 and TMPRSS2 in H1299 cells, suggests the potential to inhibit SARS-CoV-2 virus entry in the human host. Overall, the results support the valorization of the local cultivation of *P. mahaleb*.

## 1. Introduction

**Figure 1:**
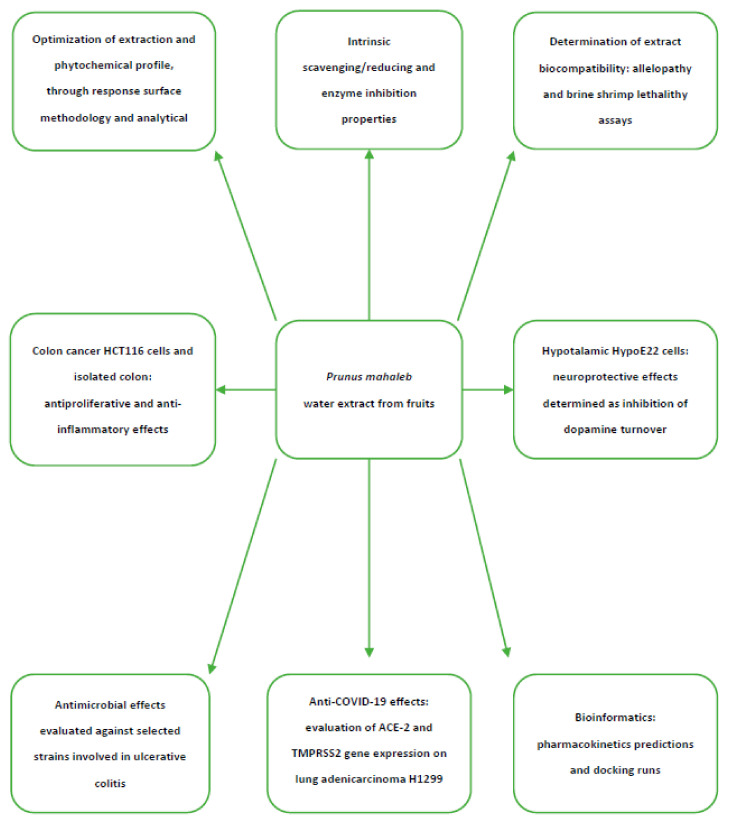
Schematic representation of the aims of the present study.

White Mahlab (*Prunus mahaleb* L.), also known as English cherry, is a deciduous tree belonging to the Rosaceae family, subfamily Prunoideae. The tree is native to the Mediterranean region, Iran, and Central Asia; however, it is also present in Eastern and Central Europe, where it prefers a hot, dry climate and well-drained soils [1]. *P. mahaleb* cherry trees are commonly used as rootstock in order to give strength and vigor to the sweet cherry; this practice is particularly diffuse in Southern Italy, in the Apulia region [2]. *P. mahaleb* blooms in mid-spring and ripens in summer, producing small, highly pigmented drupes with a diameter between 8–10 cm, and is not currently used for fresh consumption due to their sour and astringent taste [3]. The color of these fruits change from green to red and finally become black when fully ripe [4]. The fruits are generally small, spherical, juicy and with a flat surface; they also display a high content of anthocyanins. The seed is egg-shaped and pointed and represents a valuable source of proteins (30.98% *w/w*) and fatty acids (40.40% *w/w*). Polyphenols have been found in the leaves, fruits, and root wood as well [5]. The plant is also used in the production of traditional fragrances, lotions, and liqueurs [6]. The traditional liqueur called “Mirinello di Torremaggiore” (Apulia, Italy), prepared via traditional hydroalcoholic maceration of the whole fruit from *P. mahaleb*, displayed an appreciable amount of phenolic compounds, particularly in the solid residues of liqueur production [6]. The kernels of the ground seeds have a characteristic bitter taste and therefore are used as flavoring agents in bagels, cakes, muffins, and in folk medicine as diuretic, antidiabetic, tonic, aphrodisiac, and expectorant agents [4]. The whole fruit displayed scavenging and reducing activity in vitro [3]. This is consistent, albeit partially, with the phenolic composition of the phytocomplex. The in vivo administration of *P. mahaleb* prevented the formation of kidney stones as well [7], whereas in vitro studies conducted on rat kidney cells did not show cytotoxicity up to 50 mg/mL concentration [8]. However, the lack of any data about the phytochemical composition of the extracts tested on kidney cells requires further studies in order to unravel concentration-dependent effects on cell viability and metabolism [8]. Additionally, multiple studies suggest that herbal extracts rich in phenolic compounds have biocompatibility limits characterized by LC_50_ values <20 mg/mL in different in vitro models [9,10]. In this context, an accurate evaluation of the tolerability of *P. mahaleb* extracts is requested. Protective effects induced by *P. mahaleb* fruits were also observed in an experimental paradigm of ulcerative colitis [11]. Specifically, the fruit extract was able to activate the nuclear factor erythroid 2-related factor 2 pathway (Nrf2), a transcription factor deeply involved in antioxidant defense [12]. Phytochemical composition in phenolic compounds and protective effects in the colon also agree with the antiproliferative and antimicrobial effects exerted by other *Prunus* species, namely *P. cerasus* [13,14]. Still in analogy with *P. cerasus* [15], the scavenging, reducing and protective effects induced by *P. mahaleb* also indicate a potential application for counteracting the burden of oxidative stress in the brain, although scientific literature is still lacking in this field. However, Bonaventura et al. [16] showed anti-neuroinflammatory effects induced by *P. cerasus* extract administration in obese mice. The consumption of *P. avium* fruits stimulated the hypothalamic leptin pathway [17] in obese mice, thus suggesting modulatory effects on energy balance control. Considering the literature data regarding *P. mahaleb* and the pharmacological studies available on different *Prunus* species, namely *P. cerusus* and *P. avium,* a multidirectional study was conducted on the water extract of *P. mahaleb,* prepared via an ultrasound-assisted method for unravelling the phytochemical composition and the limits of biocompatibility, through the use of different toxicological paradigms, and pharmacological properties, in terms of anti-inflammatory and neuromodulatory effects. The water extract was chosen to imitate traditional home-made preparations, namely infusions and decoctions, which may not only be effective and safe due to long-term use, but may also be a novel strategy for the improvement of local botanical chains [18]. Specifically, the experimental conditions for the preparation of the water extract were optimized through the response-surface methodology (RSM) [9], a validated in silico model for predicting ab initio the optimal conditions for the plant material extraction. In this context, the RSM approach was applied for optimizing the extraction yield of total phenol and flavonoids, which were assayed via both colorimetric and high performance liquid chromatography coupled to a diode array and mass spectrometer (HPLC-DAD-MS). Colorimetric assays were also performed for measuring intrinsic scavenging and reducing and enzyme inhibition (anti-glucosidase, anti-amylase, anti-cholinesterase, and anti-tyrosinase) properties. The enzyme inhibition effects were investigated through a docking approach as well. For docking runs, we considered the prominent phenolic compounds identified and quantified in the extract. Further bioinformatics analyses were conducted for predicting the pharmacokinetic properties of these phytochemicals, especially the capability to cross the blood brain barrier that was considered as a cornerstone for supporting the reported evaluation of the extract protective effects in hypothalamic HypoE22 cells challenged with hydrogen peroxide and added to a cell medium as a pro-oxidant stimulus. In this context, the capability of *P. mahaleb* water extract in contrasting the hydrogen peroxide-induced turnover of dopamine [19] was evaluated. Considering the potential application of *P. mahaleb* in ulcerative colitis, we also investigated the protective effects of the water extract of *P. mahaleb* on isolated mouse colon specimens challenged with lipopolysaccharide (LPS) to simulate in this ex vivo model the burden of oxidative stress and inflammation occurring in ulcerative colitis [20]. The gene expression of cycloxygenase-2 (COX-2) and tumor necrosis factor-α (TNFα), deeply involved in colon inflammation [21], were measured. Additionally, the present study considered the evaluation of the gene expression of vascular endothelial growth factor A (VEGFA) and hypoxia-inducible factor 1α (HIF1α), which are well-known angiogenetic factors playing a pivotal role in the inflammatory to cancer transition in different tissues, including the colon [22]. The human colon cancer HCT116 cell line was also exposed to the present water extract in order to explore eventual antiproliferative effects. In this regard, it is sensitive to highlight the potential efficacy of an adequate intake of fruit as a strategy to prevent the onset of colorectal cancer [23]. Consistently with the evaluation of extract effects on colon pro-inflammatory biomarkers, we investigated the bacteriostatic properties of *P. mahaleb* water extract against multiple pathogen bacterial strains, namely *Escherichia coli, Pseudomonas aeruginosa,* and *Staphylococcus aureus,* which are involved in ulcerative colitis [24,25,26]. Finally, considering the very recent interest in studying natural compounds and raw extracts as anti-COVID-19 agents [27,28,29], in the present study we exposed the human H1299 lung adenocarcinoma cell line to the *P. mahaleb* extract in order to measure the gene expression of angiotensin-converting enzyme 2 (ACE2) and transmembrane protease serine 2 (TMPRSS2), which are known to play a master role in mediating SARS-CoV-2 virus entry in the human host [30,31]. For convenience, the aims of the present research are schematically summarized in Figure 1, whereas the results support the use of *P. mahaleb* fruits as sources of natural compounds with promising application in treating infectious and inflammatory diseases.

## 2. Results and Discussion

### 2.1. Phytochemical Analysis

**Figure 2:**
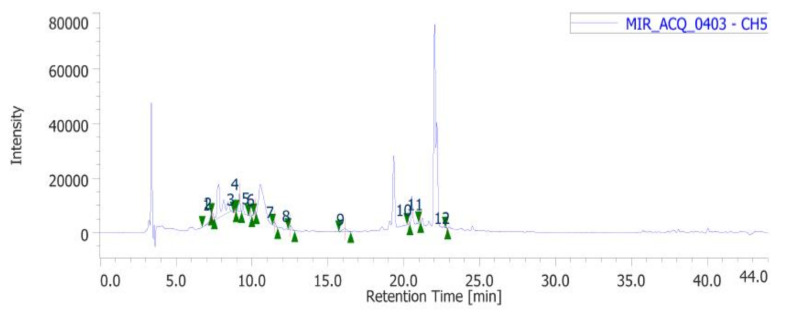
Chromatographic analysis of *Prunus mahaleb* L. phenolic compounds. The chromatographic analysis confirmed the presence of different phytochemicals, namely gallic acid (peak #2), catechin (peak #5), chlorogenic acid (peak #6), epicatechin (peak #7), caffeic acid (peak #8), chicoric acid (peak #9), coumaric acid (peak #10), ferulic acid (peak #11), and rutin (peak #12).

**Figure 3:**
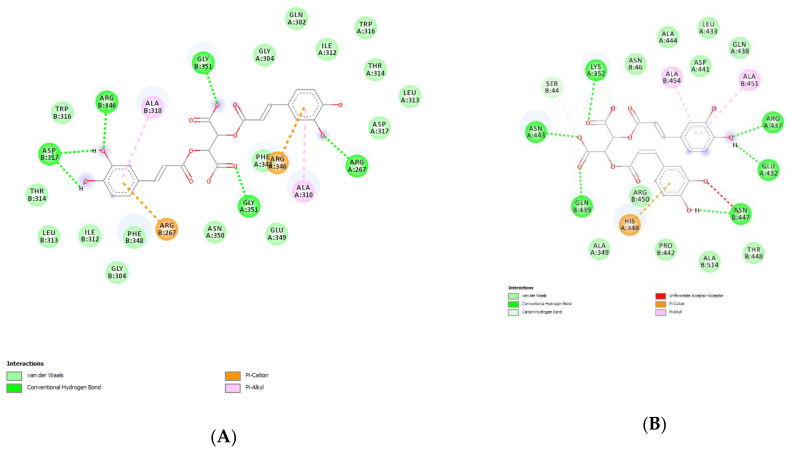

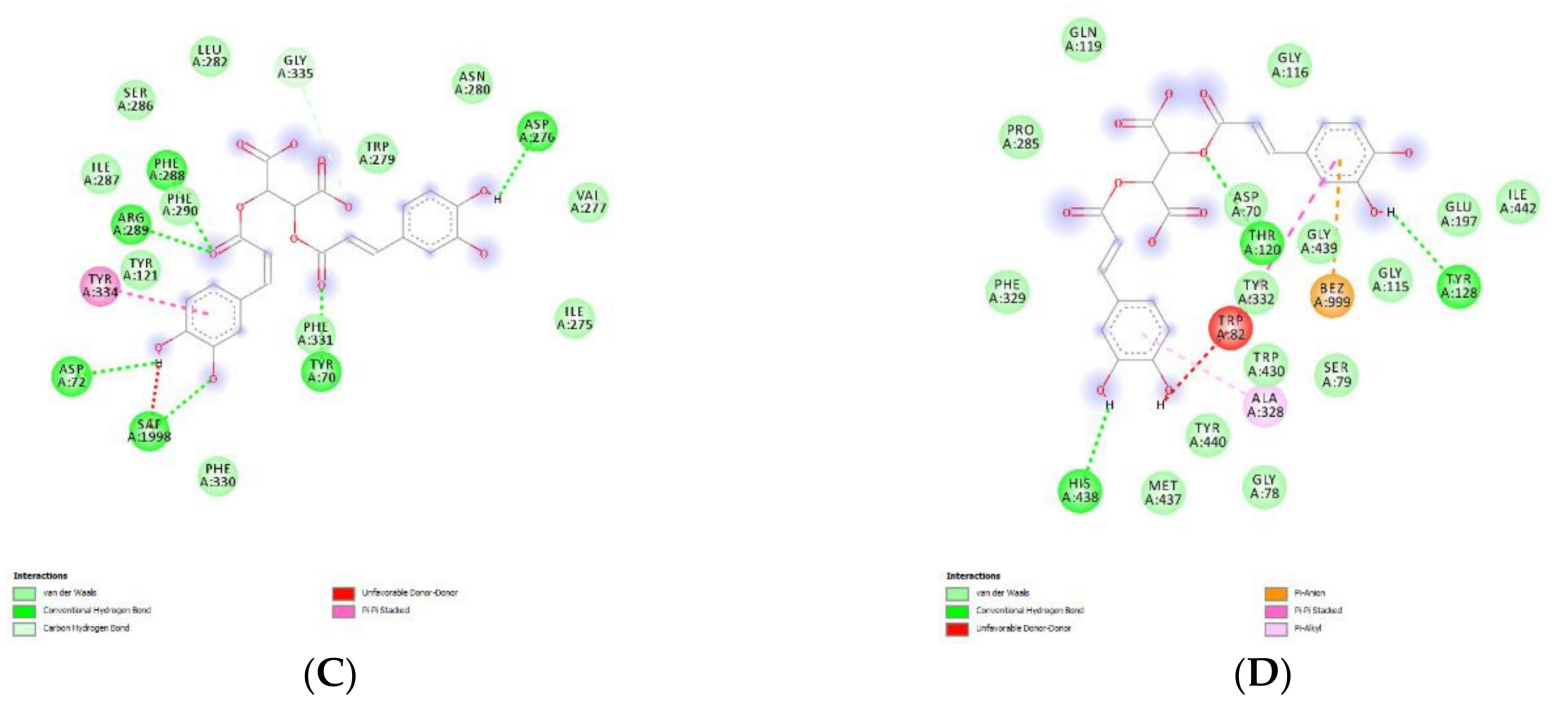
(**A**) Putative interactions between chicoric acid and α-amylase (PDB: 1XV8). Free energy of binding (ΔG) and affinity (Ki) are −9.2 kcal/mol and 0.2 µM, respectively. (**B**) Putative interactions between chicoric acid and α-glucosidase (PDB: 3WY1). Free energy of binding (ΔG) and affinity (Ki) are −8.8 kcal/mol and 0.4 µM, respectively. (**C**) Putative interactions between chicoric acid and AChE (PDB: 1GQR). Free energy of binding (ΔG) and affinity (Ki) are −8.4 kcal/mol and 0.7 µM, respectively. (**D**) Putative interactions between chicoric acid and BChE (PDB: 1GQR). Free energy of binding (ΔG) and affinity (Ki) are −9.5 kcal/mol and 0.1 µM, respectively.

In the present study, the water extract of *P. mahaleb* was analyzed for the determination of phenolic compounds. Specifically, colorimetric assays were carried out for measuring the levels of total phenols, flavonoids, and tannins. The operative conditions for the extractive procedure and experimental data of secondary metabolites quantification are described in paragraph 4.2. The total content of these metabolites in the extract is also consistent with the literature [32] and the scavenging and reducing activities depicted in Table 1. The extract also showed inhibitory effects against different enzymes, namely AChE, BChE, tyrosinase, α-amylase and α-glucosidase (Table 2). The IC_50_ values (1.28–3.44 mg/mL) related to the enzyme inhibition properties are similar to those measured for the investigation of the intrinsic scavenging and reducing properties (0.97–2.76 mg/mL). This analogy further corroborates previous studies highlighting the tight relationships between antiradical and enzyme inhibition properties [33]. Considering that these enzymes are key targets in the pharmacotherapy of neurodegenerative diseases, type 2 diabetes, and hyperpigmentation [34,35,36], and all characterized by an increased burden of inflammation and oxidative stress, the present data suggest the rationale for testing the *P. mahaleb* water extract in experimental paradigms simulating these disorders. Furthermore, considering that the observed biological properties of the extract are related, albeit partially, to the presence of phenolic compounds [37,38], a quantitative determination of selected phenols and flavonoids was conducted via HPLC-DAD-MS (Figure 2). The chromatographic analysis confirmed the presence of different phytochemicals, namely gallic acid (peak #2), catechin (peak #5), chlorogenic acid (peak #6), epicatechin (peak #7), caffeic acid (peak #8), chicoric acid (peak #9), coumaric acid (peak #10), ferulic acid (peak #11) and rutin (peak #12). Among identified compounds, the prominent were catechins and chicoric acid, which were present in the extract (20 mg/mL extract solution) in the concentration range of 2.34–3.00 µg/mL, corresponding to 4.74–9.66 µM. According to docking runs, these compounds may also be responsible, albeit partially, for the enzyme inhibitory effects shown by the extract toward AChE, BChE, tyrosinase, α-amylase, and α-glucosidase. Specifically, the putative affinities of the aforementioned phenolic compounds against the tested enzymes were in the range of 0.2–8.9 µM, therefore within the concentration interval shown by chromatographic analysis. Intriguingly, chicoric acid showed sub-micromolar affinity toward cholinesterases, α-amylase, and α-glucosidase. As depicted in Figure 3, hydrogen bond and pi interactions were mostly responsible for the calculated putative affinities. This compound was also recently identified in the fruit extract of *P. spinosa,* where its relative amount is comparable to those of catechin and epicatechin [39]. Additionally, other edible plants, including *Ocimum basilicum*, *Lactuca sativa L., Taraxacum officinale*, and *Cichorium intybus* were reported as sources of chicoric acid [40]. Chicoric acid showed multiple promising pharmacological applications, among which are the reduction of the viability of colon cancer HCT116 cells and antimicrobial and neuroprotective effects [41,42,43]. Regarding the neuroprotective effects, in silico predictions yielded by the bioinformatics platform ADMETPrediction, but also experimental data by Wang and colleagues [44], indicated the capability of chicoric acid to cross the blood brain barrier. Therefore, the inclusion of herbal extracts containing chicoric acid in experimental paradigms aiming to explore neuroprotective effects seems to be rational. Finally, chicoric acid, but also other caffeic acid derivatives, including caftaric acid, were recently described as promising natural compounds for counteracting the COVID-19 pandemic [45]. In this context, the pharmacological study described below focused on unravelling peripheral and central protective effects, antimicrobial, and also anti-COVID-19 properties by the water extract of *P. mahaleb.*

### 2.2. Toxicological and Pharmacological Studies

#### 2.2.1. Eco-Toxicological Assays

**Figure 4:**
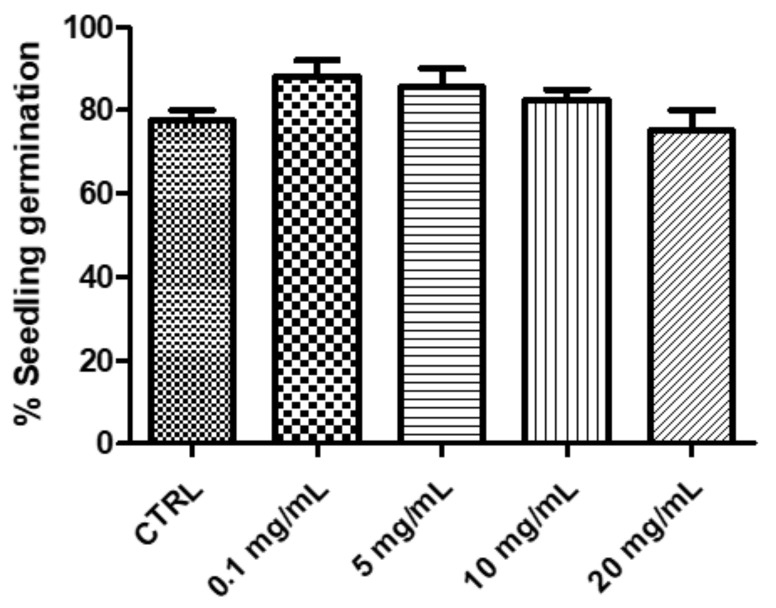
Null effect induced by *P. mahaleb* L. water extract (0.1–20 mg/mL) on the seedling germination of the lettuce variety Lollo bionda.

**Figure 5:**
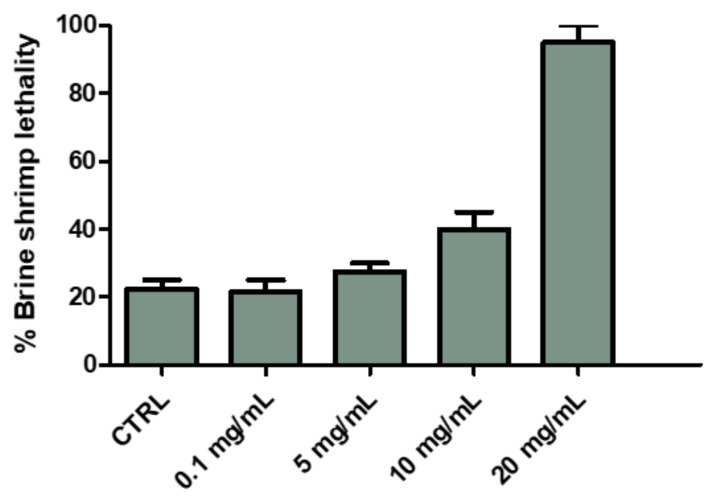
Effect of *P. mahaleb* L. water extract (0.1–20 mg/mL) on *Artemia salina* viability (brine shrimp lethality test).

The biological activity of *P. mahaleb* extract was formerly evaluated through allelopathy assay, a validated pharmacognostic test for discriminating herbal extract phytotoxicity. Particularly, the extract effects on the germination and elongation of the seeds of the lettuce cultivar Lollo bionda were tested in the concentration range 0.1–20 mg/mL. After challenging the seeds with the extract, we did not find any significant alteration on the germination process; however, at the lowest tested concentration we observed a significant root elongation (>10% compared with untreated CTR group: Figure 4). This was considered as a signal of biocompatibility; conversely, a further independent eco-toxicological assay, namely the *Artemia salina* (brine shrimp) lethality test, was performed for confirming the biocompatibility limits of the water extract. This latter test is widely used, as an alternative toxicological model, for predicting toxicity limits in eukaryotic cells. In this regard, the shrimps nauplii were exposed for 24 h to the extract (0.1–20 mg/mL). In agreement with the allelopathy assay, the extract was well-tolerated by the shrimp, with LC_50_ value > 10 mg/mL (Figure 5). Considering this result, an extract concentration at least ten-fold lower (1 mg/mL) was selected for the subsequent pharmacological assays on cell cultures and isolated mouse tissues.

#### 2.2.2. Anti-Inflammatory Effects in the Colon and Antimicrobial Properties

**Figure 6:**
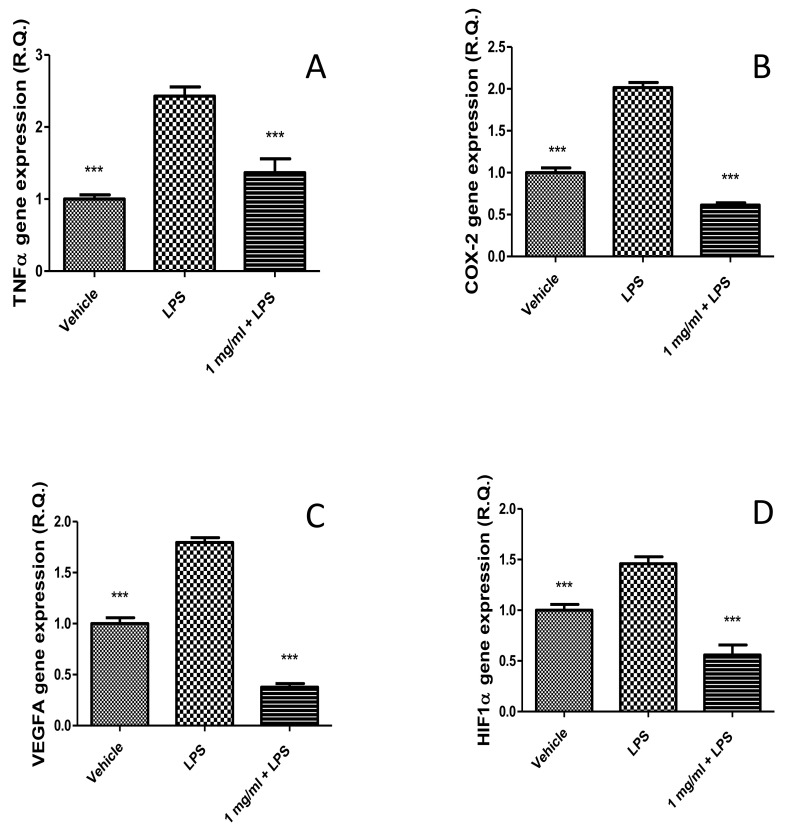
Inhibitory effects induced by *Prunus mahaleb* L. water extract (1 mg/mL) on LPS-induced upregulation of TNFα (**A**), COX-2 (**B**), VEGFA (**C**), and HIF1α (**D**) gene expression in isolated mouse colon. ANOVA, *p* < 0.0001; *** *p* < 0.001 vs. respective LPS group.

**Figure 7:**
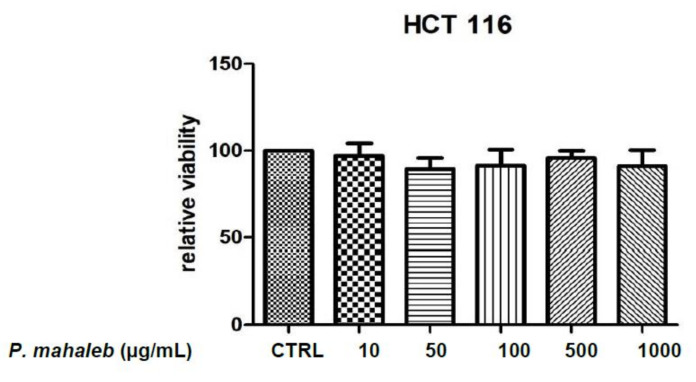
Null effect of *Prunus mahaleb* L. water extract (10–1000 µg/mL) on human colon cancer HCT116 cell viability.

After defining the limits of biocompatibility, the extract (1000 µg/mL) was tested on isolated mouse colon specimens challenged with LPS (10 µg/mL) in order to induce the burden of oxidative stress and inflammation occurring in ulcerative colitis [20,46]. In the present ex vivo experimental model, the extract was able to prevent the gene expression up-regulation of TNFα and COX-2 (Figure 6A,B), thus indicating anti-inflammatory effects in the colon that are consistent with literature data [11]. However, in the study by Ferramosca et al. [11], the evaluation of *P. mahaleb* extract on biochemical pathways involved in inflammatory responses was conducted on the liver, and the results were related to the morphological changes in the mouse colon. Therefore, our study is the first to evaluate the direct effects of *P. mahaleb* extract on pro-inflammatory biomarkers in this tissue. Additionally, we also measured the gene expression of HIF1α and VEGFA, following extract treatment. These proteins are well-known angiogenetic factors deeply involved in the inflammatory to cancer transition in different tissues, including the colon [22]. The blunting effects induced by the extract on the LPS-induced gene expression of both (Figure 6C,D) further strengthen the importance of *P. mahaleb* fruits as a source of natural compounds with protective effects in the colon. Considering the inhibition of the gene expression of all tested biomarkers that are not only involved in colon inflammation, but also in tumorigenesis [47,48,49,50], we exposed human colon cancer HCT116 cells to scalar concentrations of the extract (10–1000 µg/mL) in order to evaluate eventual antiproliferative effects on this colon cancer cell. Unfortunately, the extract did not exert any effect on HCT116 cell viability (Figure 7). Currently, the lack of efficacy as an antiproliferative agent partly agrees with literature data suggesting a good grade of tolerability of isolated cells after exposure to *P. mahaleb* extracts, with IC_50_ values higher [8] compared to the concentration range employed in the present study. Nevertheless, previous studies [8] did not report the phytochemical composition, thus making a difficult direct comparison with our data. The water extract was also tested for investigating antibacterial effects against pathogen strains involved in ulcerative colitis, namely *Escherichia coli*, *Pseudomonas aeruginosa*, and *Staphylococcus aureus* [24,25,26]. The antimicrobial assays demonstrated that the water extract of *P. mahaleb* has low antibacterial activity compared to the MIC values of the reference antibacterial drug ciprofloxacin (Table 3). However, the MIC values were in the range of biocompatibility and anti-inflammatory activity showed by the extract; thus, further suggesting its capability in exerting protective effects in the colon, with promising phytotherapy applications in the management of colon inflammatory conditions.

#### 2.2.3. Neuroprotective Effects

**Figure 8:**
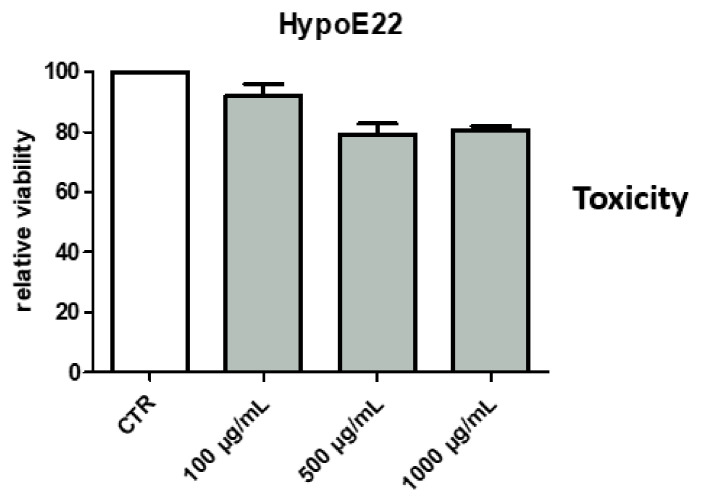
Null effect of *Prunus mahaleb* L. water extract (10–1000 µg/mL) on hypothalamic HypoE22 cell viability.

**Figure 9:**
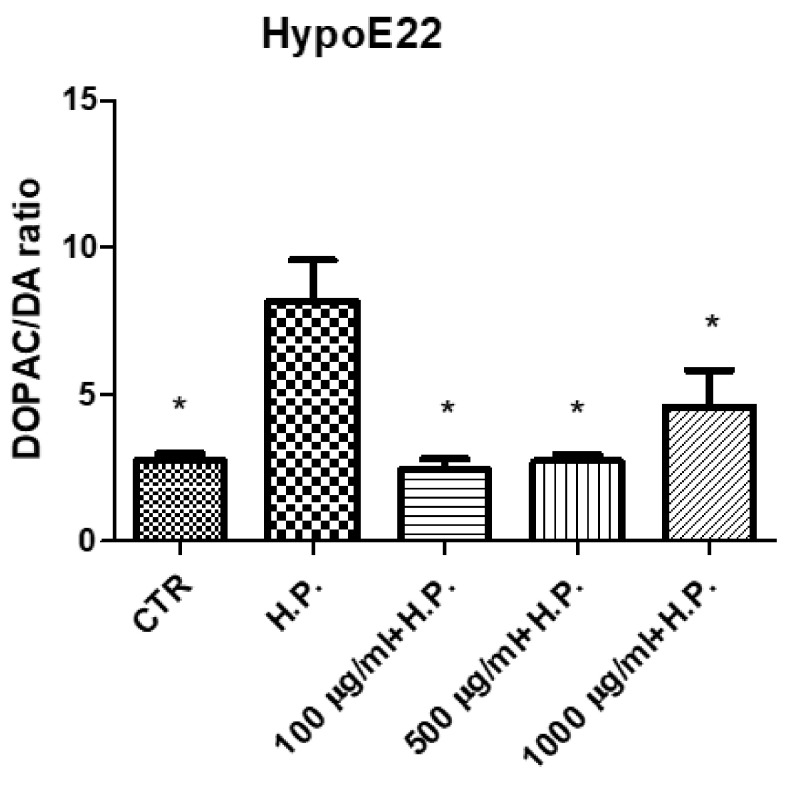
Inhibitory effects induced by *Prunus mahaleb* L. water extract (100–1000 µg/mL) on hydrogen peroxide (H.P.)-induced DA turnover (DOPAC/DA ratio). ANOVA, *p* < 0.001; * *p* < 0.05 vs. H.P. group.

**Figure 10:**
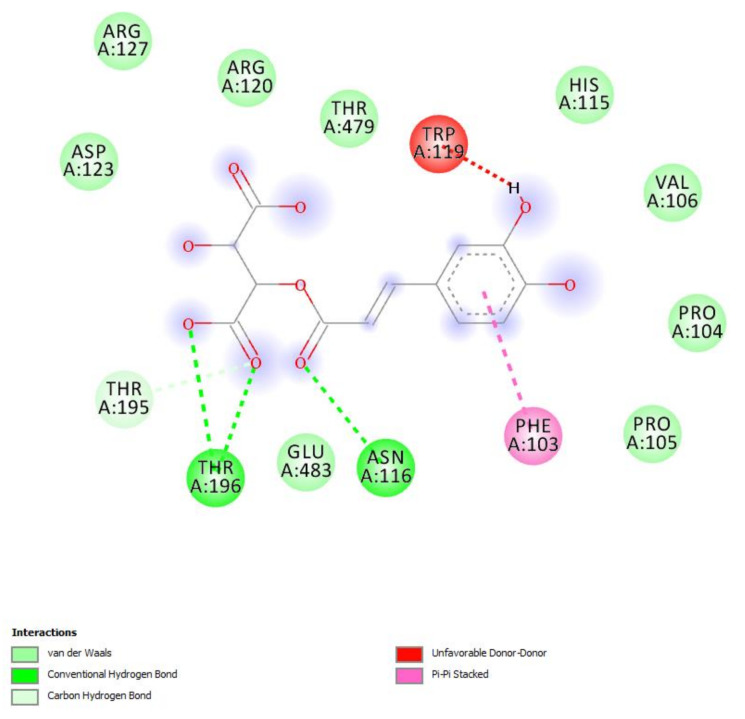
Putative interactions between chicoric acid and MAO-B (PDB: 1GOS). Free energy of binding (ΔG) and affinity (Ki) are −7.6 kcal/mol and 2.3 µM, respectively.

*P. mahaleb* water extract was also assayed on hypothalamic HypoE22 cells in order to explore potential neuroprotective effects. As shown by the MTT viability test, the extract was well-tolerated by the cell line, with % cell viability > 70% at all tested concentrations (100–1000 µg/mL) (Figure 8); thus, further confirming the good tolerability profile of the present extract. Additionally, when cells were exposed to the pro-oxidant stimulus constituted by hydrogen peroxide 300 µM, the extract was effective in preventing the turnover of DA, measured as DOPAC/DA ratio (Figure 9). DOPAC/DA ratio is also a valuable index of monoamine oxidase-B (MAO-B) activity [51], while different herbal extracts were able to prevent brain DA degradation induced by pro-oxidant stimuli [10,52]. In the case of the *P. mahaleb* extract, the presence of chicoric acid, with demonstrated capability to cross the blood brain barrier, is sensitive to hypothesize future phytotherapy applications of the *P. mahaleb* fruit within herbal products to contrast the degradation of DA occurring in Parkinson’s disease. In this regard, there is an increasing interest in studying new herbal formulations, including medicinal plants with multiple neuroprotective mechanisms, including the reduction of DA turnover [52,53,54]. The putative micromolar affinity of the chicoric acid toward MAO-B (Figure 10) is a further stimulus to deepen our knowledge about the neuroprotective effects of this plant that also add not only to the anti-neuroinflammatory effects induced by other *Prunus* species [16], but also to the capability of the sole chicoric in improving neuron survival and reduced memory impairment in different experimental models of neuroinflammation [42,55].

#### 2.2.4. Protective Effects against COVID-19 Infection

**Figure 11:**
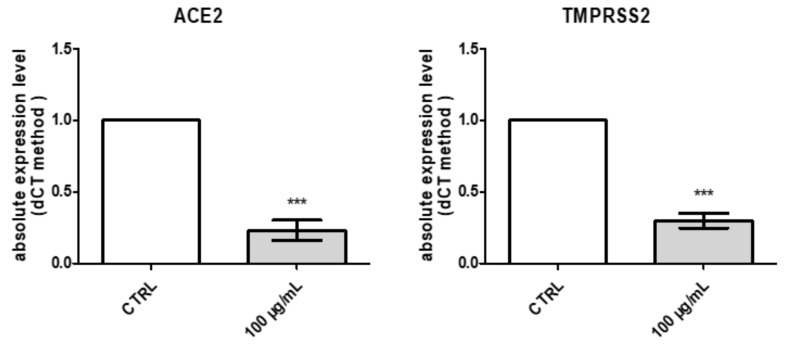
Inhibitory effects induced by *Prunus mahaleb* L. water extract (100–1000 µg/mL) on ACE2 and TMPRSS2 gene expression in H1299 lung adenocarcinoma cells. *** *p* < 0.01 vs. respective CTR group.

**Figure 12:**
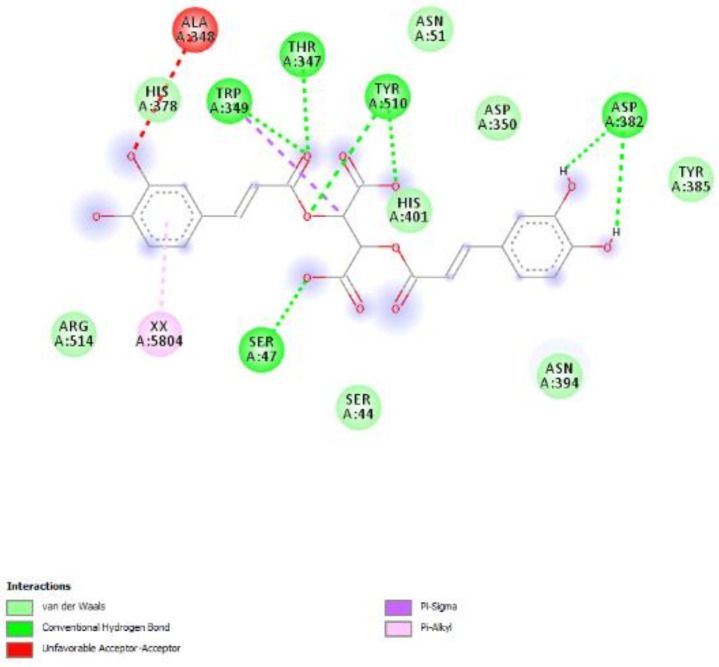
Putative interactions between chicoric acid and ACE2 (PDB: 1R4L). Free energy of binding (ΔG) and affinity (Ki) are −7.5 kcal/mol and 3.2 µM, respectively.

Finally, considering the very recent interest in studying natural compounds and herbal products as anti-COVID-19 agents [27,28], *P. mahaleb* water extract was also investigated in an in vitro model constituted by H1299 lung adenocarcinoma cells, which were reported to express ACE2 and TMPRSS2 [29]. These proteins are deeply involved in mediating SARS-CoV-2 virus entry in the human host [30,31], and the present extract was able to downregulate the gene expression of both proteins at the concentration of 100 µg/mL (Figure 11). Considering the results of the quantitative analysis conducted on the extract, but also the in silico studies carried by Adem and colleagues that pointed to chicoric as promising natural compound for counteracting the COVID-19 pandemic, we also evaluated the affinity of this phytochemical toward ACE2. Regarding this docking approach, we did not consider the putative interactions with TMPRSS2; indeed, the TMPRSS2 structure is not available, and any in silico prediction currently available in the literature [56] are based on hepsin, which is used in view of its homology with TMPRSS2. The results of the docking yielded micromolar affinity of chicoric acid toward ACE2 (Figure 12), thus partly substantiating the pattern of gene expression in H1299 cells. These promising results support future studies that may be driven with the aim to include the present extract in protecting devices for preventing the SARS-CoV-2 virus entry into the human host.

## 4. Materials and Methods

### 4.1. Plant Material and Reagents

The plant material consisted of fresh fruits manually collected from *Prunus mahaleb* L. and cultivated in the surrounding of Torremaggiore (Apulia, Italy). The *Prunus* plantation was specifically dedicated to collecting fruits for the preparation of the local traditional liqueur called “Mirinello” that is identified as a traditional agrifood product by the Italian Ministry for Agricultural Policies (GU—Serie Generale n. 48 del 26-2-2021 s. ord n 15). It can be considered a modern example of plant domestication. The plant is widely distributed, but for the production of the traditional liqueur, the fruits are collected only from cultivated plants. The origin of this practice is not documented, but still today, for the implementation of plantation, shoots from old samples are collected and grafted on wild *P. mahleb* rootstock. Fruits at the full ripening stage were manually collected and frozen at −80 °C within 6 h. After 24 h, the fruits were stored in plastic sealed bags in the dark at −20 °C until used to perform phytochemical and biological assays. Plant identity was confirmed botanically and morphologically by the co-author Prof. Luigi Menghini. The sample used was an aliquot of collection destined to the industrial production of liqueur and was kindly supplied by Mirinello Liquori S.r.l. (Torremaggiore, Apulia, Italy). Phenolic compound standards were purchased from Sigma-Aldrich (Milano, Italy).

### 4.2. Response Surface Methodology (RSM)

The sample of fruits was weighed using a Precisa XT220A balance (Micro Precision Calibration Inc., Grass valley, CA, USA) in 50 mL Falcon tubes and then immediately homogenized together with the extraction solvent using a T25 digital Ultra-Turrax tissue homogenizer (IKA, Staufen, Germany) for 30 s at 10,000 g. This treatment partially uniformed the grain size; thus, a better extraction could be performed. Subsequently, ultrasound-assisted water extraction (UAE) of the homogenate was conducted. The sample tube with the mixture was placed in a Trans-sonic T460 ultrasonic bath (Elma, Singen, Germany). The operative conditions for the extraction were optimized through response surface methodology (RSM). A four-factors Box-Behnken design was defined to investigate the effects of parameters such as time, temperature, solid/liquid ratio and percentage of ethanol on UAE of *P. mahaleb* fruits. The effects of independent variables were evaluated as total phenol content (TPC), total flavonoid content (TFC), and total tannin content (TTC). The operative conditions such as extraction method (UAE) were selected on the basis of a previous study [57] while water and ethanol were selected as solvent for extraction due to their food-use compatibility. The range applied for selecting independent variables are detailed in Table 4.

According to the experimental design, a set of experiments of factorial design at three levels and four factors, with a total of twenty-seven runs, including three replicates at the central point, were applied to evaluate the curvature model, as reported in Table 5. Considering that *P. mahaleb* is traditionally used in the preparation of liqueurs through hydroalcoholic maceration, the RSM also considered the comparison between the water extract and hydroalcoholic solutions. The surface analysis and analysis of variance (ANOVA) to define and optimize the Box-Behnken experimental conditions were conducted with Minitab 16 software. The predicted conditions reported at line 18 of Table 5 permitted to obtain the best results in terms of yield in total phenols (TPC) and total tannins (TTC). Specifically, the best conditions for the ultrasound-assisted extraction in water were: time = 32.4 min; TEMP = 52.5 °C; and frequency = 30 kHz. Further details about RSM are included as Supplementary Materials.

### 4.3. Scavenging and Reducing and Enzyme Inhibition Properties

Intrinsic scavenging and reducing properties of the extracts were determined through colorimetric assays [58]. Additionally, extracts were assayed for evaluating enzyme inhibition effects toward tyrosinase, α-amylase, α-glucosidase, and cholinesterases. Detailed protocols were reported in previous studies [58].

### 4.4. Phenolic and Flavonoid Determination: Colorimetric and HPLC-DAD-MS Analyses

The colorimetric measurement of total phenolic, flavonoid, and tannin levels was conducted according to a recent study [59]. Standards, namely gallic acid (GA) for phenolics, rutin (RU) for flavonoids, and tannic acid for tannins were used to explain the results. The identification and quantification of selected phenolic compounds were conducted through HPLC-DA-MS analysis. The HPLC apparatus consisted of a two PU-2080 PLUS chromatographic pump, a DG-2080-54 line degasser, a mix-2080-32 mixer, UV, diode array (DAD) and detectors, a mass spectrometer (MS) detector (expression compact mass spectrometer (CMS), Advion, Ithaca, NY 14850, USA), an AS-2057 PLUS autosampler, and a CO-2060 PLUS column thermostat (all from Jasco, Tokyo, Japan). Integration was performed by ChromNAV2 Chromatography software. Before injecting in the HPLC apparatus, the extract was centrifuged at 5000 rpm for 15 min, and supernatant diluted at 10 mg/mL. Water extract (10 mg/mL) was analyzed for phenol quantitative determination using a reversed-phase HPLC–DAD-MS in gradient elution mode, in agreement with literature data [60]. The separation was conducted within the 32 min of the chromatographic run, starting from the following separation conditions: 0.1% formic acid, 95% water, and 5% methanol. The separation was performed on an Infinity lab Poroshell 120 reverse phase column (C18, 150 mm × 4.6 mm i.d., 2.7 µm) (Agilent Santa Clara, CA, USA). Column temperature was set at 30 °C. Quantitative determination of phenolic compounds was performed via DAD detector. The extract was also qualitatively analyzed with MS detector in negative ion mode (vanillic acid, ferulic acid, and naringenin) and positive ion mode (rutin). MS signal identification was realized through comparison with standard solutions and MS spectra present in the MassBank Europe database. The list of compounds analyzed and the wavelengths and the m/z ratio for their determination are listed in Table 6. Quantification was done through seven-point calibration curves, with linearity coefficients (R2) > 0.999, in the concentration range of 2–140 µg/mL. The limits of detection were lower than 1 µg/mL for all assayed analytes. The area under the curve from HPLC chromatograms was used to quantify the analyte concentrations in the extract.

### 4.5. Eco-Toxicological Profile: Allelopathy and Artemia salina (brine shrimp) Lethality Assays

Allelopathy bioassay was carried on the seeds of the commercial lettuce variety Lollo bionda because of its fast germination rate and high sensitivity. The detailed procedure was conducted as previously reported [10]. Seeds were treated with scalar *P. mahaleb* extract concentrations (0.1–20 mg/mL) and considered germinated for observed root length ≥ 1 mm, after the third day of treatment. *Artemia salina* cysts were cultivated in oxygenated artificial sea water (1 g cysts/L). After 24 h, brine shrimp larvae were gently transferred with a pipette in 6 well plates containing 2 mL of the extract at different concentrations (0.1–20 mg/mL) in artificial sea water. The detailed protocol is reported in our previous paper [10].

### 4.6. Human Colon Cancer HCT116 Cells: Evaluation of Antiproliferative Effects

Human colon cancer-derived HCT116 cells were cultured in DMEM (Euroclone) supplemented with 10% (*v*/*v*) heat-inactivated fetal bovine serum and 1.2% (*v*/*v*) penicillin G/streptomycin in a 75 cm2 tissue culture flask (n = 5 for individual culture flasks for each condition). The cultured cells were maintained in a humidified incubator with 5% CO_2_ at 37 °C. For cell differentiation, HCT116 cell suspension at a density of 1 × 106 cells/mL was treated with various concentrations (10, 50, and 100 ng/mL) of phorbol myristate acetate (PMA, Fluka) for 24 h or 48 h (induction phase). Thereafter, the PMA-treated cells were washed twice with ice-cold pH 7.4 phosphate buffer solution (PBS) to remove PMA and non-adherent cells, whereas the adherent cells were further maintained for 48 h (recovery phase). Morphology of cells was examined under an inverted phase-contrast microscope. To assess the basal cytotoxicity of water extract, a viability test was performed on 96 microwell plates, using 3-(4,5-dimethylthiazol-2-yl)-2,5-diphenyltetrazolium bromide (MTT) test. Cells were incubated with extracts (ranging in the concentration 10–1000 μg/mL) for 24 h. A total of 10 μL of MTT (5 mg/mL) was added to each well and incubated for 3 h. The formazan dye formed was extracted with dimethyl sulfoxide and absorbance was recorded as previously described [38]. Effects on cell viability were evaluated in comparison to the untreated control group.

### 4.7. Isolated Mouse Colon Specimens: Evaluation of Anti-Inflammatory Effects

Fifteen male adult mice were housed in plexiglass cages (40 cm × 25 cm × 15 cm), two rats per cage, in climatized colony rooms (22 ± 1 °C; 60% humidity), on a 12 h/12 h light/dark cycle (light phase: 07:00–19:00 h), with free access to tap water and food, 24 h/day throughout the study, with no fasting periods. Mice were fed a standard laboratory diet (3.5% fat, 63% carbohydrate, 14% protein, 19.5% other components without caloric value; 3.20 kcal/g). Housing conditions and experimentation procedures were strictly in accordance with the European Union ethical regulations on the care of animals for scientific research. According to the recognized ethical principles of “Replacement, Refinement and Reduction of Animals in Research”, colon specimens were obtained as residual material from vehicle-treated rats randomized in our previous experiments and approved by a local ethical committee (University “G. d’Annunzio” of Chieti-Pescara) and the Italian Health Ministry (Italian Health Ministry authorization N. F4738.N.5QP). Mice were sacrificed by CO_2_ inhalation (100% CO_2_ at a flow rate of 20% of the chamber volume per min) and colon specimens were immediately collected and maintained in a humidified incubator with 5% CO_2_ at 37 °C for 4 h, in RPMI buffer with added bacterial LPS (10 μg/mL) (incubation period). During the incubation period, tissues were treated with the sub-toxic concentration of water extract (1000 μg/mL). Tissue specimens and supernatants were collected for gene expression and chromatographic analyses, respectively, of pro-inflammatory biomarkers, as detailed below.

### 4.8. Hypothalamic HypoE22 Cells: Evaluation of Neuroprotective Effects

HypoE22 cells were purchased from Cedarlane Cellution Biosystem and cultured in DMEM (Euroclone) supplemented with 10% (*v*/*v*) heat-inactivated fetal bovine serum and 1.2% (*v*/*v*) penicillin G/streptomycin in a 75 cm^2^ tissue culture flask (*n* = 5 individual culture flasks for each condition). The culture conditions and the viability 3-(4,5-dimethylthiazol-2-yl)-2,5-diphenyltetrazolium bromide (MTT) test were performed as previously described [9]. Effects of the extract (100–1000 µg/mL) on cell viability were evaluated in comparison to the untreated control group, constituted by either vehicle or hydrogen peroxide (H.P.) 300 µM stimulus.

### 4.9. Human H1299 Lung Adenocarcinoma Cell Line: Anti-COVID-19 Effects

The human H1299 lung adenocarcinoma cell line was cultured with an RMPI-16140 medium supplemented with 10% heat-inactivated fetal bovine serum, 100 U/mL penicillin, and 100 µg/mL streptomycin. Cells were incubated at 37 °C with 5% CO_2_. The H1299 cell line was purchased from ATCC (Manassas, VA, United States). In the experiments, cells were seeded into six well culture plates, kept ad concentration of 2 × 10^5^ cells/mL, and after 24 h, were treated with different concentrations of the extract (500–1000 µg/mL) for 24 h. After cell stimulation, total mRNA was extracted for the evaluation of ACE2 and TMPRSS2 gene expression.

### 4.10. Gene Expression Analysis

Gene expression of TNFα, COX-2, VEGF, HIF1α, ACE2, and TMPRSS2 was conducted as previously reported [60]. Briefly, after extraction through the TRI Reagent, total RNA was reverse transcribed using High Capacity cDNA Reverse Transcription Kit (ThermoFischer Scientific, Waltman, Massachusetts, USA). Gene expression was determined by quantitative real-time PCR using TaqMan probes obtained from ThermoFischer Scientific (Waltman, Massachusetts, USA). β-actin was used as the housekeeping gene. The analysis of data was conducted with the Sequence Detection System (SDS) software version 2.3 (ThermoFischer Scientific, Waltman, Massachusetts, USA). A detailed description of the experimental protocol is reported in a previous paper of ours [20].

### 4.11. Quantitative Determination of Dopamine (DA), Dihydroxyphenilacetic Acid (DOPAC)

DA and DOPAC levels were analyzed through an HPLC apparatus consisting of a Jasco (Tokyo, Japan) PU-2080 chromatographic pump and an ESA (Chelmsford, MA, USA) Coulochem III coulometric detector, equipped with a microdialysis cell (ESA-5014b) porous graphite working electrode and solid state palladium reference electrode. The detailed description of the chromatographic analysis is fully described in our previous study [61].

### 4.12. Antibacterial Effects

In vitro antimicrobial effects of the water extract from *P. mahaleb* were assessed against three bacterial strains (CLSI M07-A9), namely *E. coli* (ATCC 10536), *P. aeruginosa* (ATCC 15442), and *S. aureus* (ATCC 6538). Detailed description of the experimental protocol is reported in our recent paper, [62].

### 4.13. Bioinformatics

*In silico* pharmacokinetics evaluations were conducted on the platforms SwissAdmePrediction and ADMETPrediction. Docking calculations were conducted through the Autodock Vina of PyRx 0.8 software, as recently described [63]. Crystal structures of target proteins were derived from the Protein Data Bank (PDB) with PDB ID as follows: 1R4L (inhibitor-bound human angiotensin-converting enzyme-related carboxypeptidase: ACE2), 513B (Tyrosinase), 1XV8 (α-amylase), 3WY1 (α-glucosidase), 1GQR (acethylcholinesterase: AchE), 309M (Butyrylcholinesterase: BChE), and 1GOS (Monoaminoxidase-B: MAO-B). Discovery studio 2020 visualizer was employed to investigate the protein–ligand nonbonding interactions.

### 4.14. Statistical Analysis

The experimental data related to in vitro and ex vivo studies were analyzed through the analysis of variance (ANOVA) followed by Newman-Keuls post hoc test. The GraphPad Prism software was employed for statistical analysis. *p* < 0.05 was considered statistically significant. The number of animals to be employed in the study was calculated using G*Power software (v3.1.9.4, University of Kiel, Kiel, Germany). The values of the study potency (1-β) and the significance level (α) were 0.8 and 0.05, respectively.

## 5. Conclusions

In conclusion, the present study explored the health potential of the water extract from the fruit of *P. mahaleb*, a wild edible plant that has been used for centuries in the liqueur tradition. The study explored the phytochemical composition in phenolic compounds, finding significant amounts of catechin and chicoric acid that may explain, albeit partially, the observed pharmacological properties, in terms of protective effects against inflammatory and infectious diseases. In this regard, the bacteriostatic effects induced on Gram+ and Gram- strains, together with the inhibition of COX-2, TNFα, HIF1α, and VEGFA suggest the potential of *P. mahaleb* water extract in contrasting the clinical symptoms related to ulcerative colitis. The inhibition of hydrogen peroxide-induced DOPAC/DA ratio, in hypothalamic neurons, indicates promising neuroprotective effects. In view of future in vivo studies to confirm this finding, it is sensitive to highlight the capability of chicoric acid to cross the blood brain barrier and its putative affinity toward MAO-B, which is deeply involved in DA turnover. Finally, but not for importance, there is the ability of the extract to downregulate the gene expression of ACE2 and TMPRSS2 in human adenocarcinoma H1299 cells. As ACE2 and TMPRSS2 are involved in SARS-CoV-2 virus entry in the human host, with the present findings, we hypothesize the inclusion of the present extract in protection devices, such as surgical masks, functioning as physical barriers against COVID-19. Overall, the results of this research point to the valorization of the local cultivation of *P. mahaleb*, an ancient botanical resource with promising health perspectives.

## Figures and Tables

**Table 1:** Scavenging/reducing properties.

Treatments	DPPH	ABTS	CUPRAC	FRAP	Chelating Ability	PBD
*P. mahaleb* water extract	1.16 ± 0.01	1.11 ± 0.03	1.68 ± 0.09	0.97 ± 0.05	1.33 ± 0.04	2.76 ± 0.09
TROLOX	0.05 ± 0.01	0.08 ± 0.01	0.11 ± 0.01	0.04 ± 0.01	nt	0.60 ± 0.02
EDTA	nt	nt	nt	nt	0.03±0.01	nt

nt: not tested. PBD: Phosphomolybdenum. Values are reported as IC_50_ (mg/mL).

**Table 2:** Enzyme inhibition properties.

Treatments	AchE	BChE	Tyrosinase	α-Amylase	α-Glucosidase
*P. mahaleb* water extract	1.53 ± 0.10	1.34 ± 0.05	1.28 ± 0.04	3.44 ± 0.14	1.35 ± 0.04
Galantamine	0.003 ± 0.0001	0.004 ± 0.0001	nt	nt	nt
Kojic acid	nt	nt	0.08 ± 0.01	nt	nt

nt: not tested. Values are reported as IC_50_ (mg/mL).

**Table 3:** Antibacterial effects by *P. mahaleb* water extract.

Treatments	Bacterial Strains	MIC (µg/mL)
*P. mahaleb water* extract	*E. coli* (ATCC 10 536)	31.49 (25–50)
*P. mahaleb water* extract	*P. aeruginosa* (ATCC 15442)	>200
*P. mahaleb water* extract	*B. cereus* (ATCC 12826)	>200
Ciprofloxacin	*E. coli* (ATCC 10536)	<0.12
Ciprofloxacin	*P. aeruginosa* (ATCC 15442)	1.23 (1.95–0.98)
Ciprofloxacin	*B. cereus* (ATCC 12826)	0.62 (0.98–0.49)

**Table 4:** Factors and relative extreme level applied to the experimental design.

Independent Variables	Levels	
	−1	1
Time (min)	5	60
Temperature (°C)	25	80
Ethanol percentage	0	100
Solid/liquid (g/mL)	0.010	0.10

**Table 5:** Experimental design matrix with coded variables and experimental data for total polyphenols, total flavonoids, and total tannins.

	Variables				Experimental Results					
Conditions	Time (min)	Temp (°C)	Ethanol %	Solid/Liquid (g/mL)	TPC	SD	TFC	SD	TTC	SD
1	5	52.5	50	0.01	0.198	0.035	0.044	0.001	0.184	0.005
2	5	52.5	50	0.1	0.429	0.018	0.148	0.003	0.469	0.053
3	60	52.5	50	0.01	0.130	0.003	0.003	0.000	0.127	0.007
4	60	52.5	50	0.1	0.658	0.023	0.197	0.006	0.591	0.032
5	32.5	25	0	0.055	0.612	0.005	0.104	0.001	0.599	0.053
6	32.5	25	100	0.055	0.236	0.011	0.086	0.002	0.206	0.013
7	32.5	80	0	0.055	0.648	0.082	0.096	0.002	0.645	0.048
8	32.5	80	100	0.055	0.263	0.007	0.099	0.003	0.250	0.046
9	32.5	25	50	0.01	0.176	0.003	0.034	0.002	0.175	0.009
10	32.5	25	50	0.1	0.447	0.013	0.119	0.001	0.437	0.024
11	32.5	80	50	0.01	0.122	0.003	0.024	0.002	0.105	0.008
12	32.5	80	50	0.1	0.749	0.082	0.252	0.002	0.589	0.033
13	5	52.5	0	0.055	0.568	0.011	0.099	0.001	0.587	0.060
14	60	52.5	0	0.055	0.597	0.015	0.104	0.003	0.616	0.068
15	5	52.5	100	0.055	0.235	0.012	0.084	0.002	0.208	0.007
16	60	52.5	100	0.055	0.243	0.002	0.084	0.002	0.182	0.006
17	32.5	52.5	0	0.01	0.164	0.002	0.028	0.001	0.147	0.016
18	32.5	52.5	0	0.1	0.769	0.008	0.143	0.002	0.812	0.038
19	32.5	52.5	100	0.01	0.078	0.005	0.019	0.001	0.105	0.034
20	32.5	52.5	100	0.1	0.311	0.017	0.104	0.002	0.248	0.005
21	5	25	50	0.055	0.466	0.008	0.141	0.002	0.354	0.027
22	60	25	50	0.055	0.455	0.012	0.133	0.002	0.291	0.009
23	5	80	50	0.055	0.367	0.041	0.100	0.003	0.368	0.017
24	60	80	50	0.055	0.421	0.019	0.122	0.005	0.394	0.008
25	32.5	52.5	50	0.055	0.374	0.012	0.123	0.005	0.386	0.026
26	32.5	52.5	50	0.055	0.531	0.016	0.161	0.001	0.332	0.012
27	32.5	52.5	50	0.055	0.527	0.030	0.149	0.002	0.313	0.026

Run sequence was conducted randomly. TPC: total polyphenols content expressed as GAE (mg/g); TFC: total flavonoids content expressed as rutin equivalents; TTC: total tannins content expressed as tannic acid equivalents.

**Table 6:** Wavelengths of quantification and retention times related to the investigated phenolic compounds.

Standard	*m/z*	Wavelengths (nm)	Retention Time (min)
Gallic acid	169.1	254	7.303
Catechin	289.3	254	9.867
Chlorogenic acid	353.31	254	10.203
Epicatechin	289.3	254	11.473
Caffeic acid	179.16	254	12.533
Chicoric acid	473.37	254	16.117
Coumaric acid	163.04	254	20.293
Ferulic acid	193.1	254	21.033
Rutin	611.5	254	22.813

## Data Availability

Not applicable.

## Supplementary Materials

Supplementary material are available online.

## Sample Availability

Samples of the compounds are not available from the authors.
