# Supplementary material for: Unravelling the Phytochemical Composition and the Pharmacological Properties of an Optimized Extract from the Fruit from Prunus mahaleb L.: From Traditional Liqueur Market to the Pharmacy Shelf
Source: Molecules. 2021 Jul 22;26(15):4422. doi: 10.3390/molecules26154422 (PMC8347645; doi:10.3390/molecules26154422)
Supplement: Supplementary file 1 [file molecules-26-04422-s001.zip › molecules-1299435-supplementary.pdf]

molecules

Supplementary Materials

Unravelling the Phytochemical Composition and the Pharmacological Properties of an Optimized Extract from the Fruit from *Prunus mahaleb* L.: From Traditional Liqueur Market to the Pharmacy Shelf

Giustino Orlando [1], Annalisa Chiavaroli [1], Sabrina Adorisio [2], Domenico V. Delfino [2], Luigi Brunetti [1], Lucia Recinella [1], Sheila Leone [1], Gokhan Zengin [3,*], Alessandra Acquaviva [1,4], Paola Angelini [5], Giancarlo Angeles Flores [5], Roberto Venanzoni [5], Simonetta Cristina Di Simone [1], Francesca Di Corpo [1], Andrei Mocan [6,7,*], Luigi Menghini [1,*] and Claudio Ferrante [1]

[1] Department of Pharmacy, Botanic Garden "Giardino dei Semplici", Università degli Studi "Gabriele d'Annunzio", via dei Vestini 31, 66100 Chieti, Italy; giustino.orlando@unich.it (G.O.); annalisa.chiavaroli@unich.it (A.C.); luigi.brunetti@unich.it (L.B.); lucia.recinella@unich.it (L.R.); sheila.leone@unich.it (S.L.); alessandra.acquaviva@unich.it (A.A.); disimonesimonetta@gmail.com (S.C.D.S.); francesca.dicorpo@studenti.unich.it (F.D.C.); claudio.ferrante@unich.it (C.F.)
[2] Section of Pharmacology, Department of Medicine and Surgery, Foligno Nursing School, University of Perugia, 06100 Perugia, Italy; adorisiosabrina@libero.it (S.A.); domenico.delfino@unipg.it (D.V.D.)
[3] Department of Biology, Science Faculty, Selcuk University, Campus, Konya 42130, Turkey; gokhanzengin@selcuk.edu.tr
[4] Veridia Italia Srl, Via Raiale 285, 65100 Pescara, Italy; alessandra.acquaviva@unich.it
[5] Department of Chemistry, Biology and Biotechnology, University of Perugia, 06100 Perugia, Italy; paola.angelini@unipg.it (P.A.); giancarlo.angelesflores@studenti.unipg.it (G.A.F.); roberto.venanzoni@unipg.it (R.V.)
[6] Faculty of Pharmacy, "Iuliu HaŢieganu" University of Medicine and Pharmacy, 8 Victor Babeş Street, 400012 Cluj-Napoca, Romania; mocan.andrei@umfcluj.ro
[7] Laboratory of Chromatography, Institute of Advanced Horticulture Research of Transylvania, University of Agricultural Sciences and Veterinary Medicine, 400372 Cluj-Napoca, Romania; mocan.andrei@umfcluj.ro
* Correspondence: gokhanzengin@selcuk.edu.tr (G.Z.); mocan.andrei@umfcluj.ro (A.M.); luigi.menghini@unich.it (L.M.)

Response surface methodology (RSM)

A set of experiments of factorial design at three levels, four factors with a total of 27 runs, including 3 replicates at the central point, were applied to evaluate the curvature model. The independent variables were plant/solvent ratio, sonication time, percentage of ethanol and temperature of sonication. The effects were evaluated as extraction of total phenolic compounds, total flavonoids content, and total tannin.

The independent variables were evaluated in a Box-Behnken response surface design as well as their interactions. In Table S1 are shown the estimated regression coefficients of the second order polynomial equations for RSM analysis of TPC, TFC and TTC extraction (uncoded) from *P. mahleb* fruits.

Regression coefficients for mean, linear, interaction and quadratic terms, were calculated respectively from the experimental results by the least squares method. Minitab 16 software was used. The ANOVA analysis was applied to evaluate the relevance of independent variables' influence and interactions ($p<0.05$). The adequacy of the model was predicted on the basis of the coefficient of determination (Rsq), the significance (p) and the lack of adjustment tests.

Table S1. Estimated regression coefficient of the second order polynomial equation for response surface methodology analysis of secondary metabolite classes extraction (uncoded).

TERM	Estimated Regression Coefficients	P	Regression p-value	R squared	Lack of fit
**Polyphenols**					
**Constant**	0,305629	0,000	0.000	95,58%	0.878
**plant/solvent**	6,33380	0,000			
**Time**	-7,49586E-04	0,285			
**Et-OH %**	0,000662442	0,000			
**Temp °C**	-0,00294133	0,425			
**plant/solvent*plant/sol-vent**	-48,7814	0,003			
**time*time**	-4,22369E-05	0,258			
**Et-OH %*Et-OH %**	-1,49100E-05	0,191			
**Temp °C*Temp °C**	-1,02918E-05	0,777			
**plant/solvent*time**	0,0599390	0,034			
**plant/solvent*Et-OH %**	-0,0413764	0,011			
**plant/solvent*Temp °C**	0,0718589	0,014			
**time*Et-OH %**	-3,83262E-06	0,868			
**time*Temp °C**	2,13299E-05	0,613			
**Et-OH %*Temp °C**	-1,68993E-06	0,942			
**Flavonoids**					
**Constant**	0,0567409	0,000	0.000	91.66%	0.450
**plant/solvent**	1,25611	0,000			
**Time**	-2,34313E-04	0,747			
**Et-OH %**	0,00143445	0,261			
**Temp °C**	-8,94473E-04	0,384			
**plant/solvent*plant/solvent**	-15,5483	0,010			
**time*time**	-1,78296E-05	0,216			
**Et-OH %*Et-OH %**	-1,58480E-05	0,002			
**Temp °C*Temp °C**	-9,36990E-06	0,506			
**plant/solvent*time**	0,0183162	0,082			
**plant/solvent*Et-OH %**	0,00334372	0,540			
**plant/solvent*Temp °C**	0,0291009	0,011			
**time*Et-OH %**	-8,20117E-07	0,926			
**time*Temp °C**	9,70260E-06	0,550			

**Et-OH %*Temp °C**	3,77745E-06	0,671			
**Tannins**			43.31	98.06%	0.555
**Constant**	0,384093	0,000			
**plant/solvent**	5,55237	0,000			
**Time**	-0,00402215	0,832			
**Et-OH %**	-0,00182722	0,000			
**Temp °C**	-0,00502846	0,059			
**plant/solvent*plant/solvent**	-17,3971	0,065			
**time*time**	1,66758E-05	0,481			
**Et-OH %*Et-OH %**	1,67833E-05	0,032			
**Temp °C*Temp °C**	2,37557E-05	0,320			
**plant/solvent*time**	0,0361055	0,045			
**plant/solvent*Et-OH %**	-0,0580370	0,000			
**plant/solvent*Temp °C**	0,0448036	0,017			
**time*Et-OH %**	-1,00000E-05	0,505			
**time*Temp °C**	2,94031E-05	0,288			
**Et-OH %*Temp °C**	-2,22222E-07	0,988			

The ANOVA analysis of the model for the polyphenol extractions from *Prunus* fruits shows that the models is significant ($p<0,05$) according to R squared and p- values for polyphenols and flavonoids and not significant for tannins. The missing of significance for the lack of fit ($p = 0.45$; $p=0,555$) in the model support the applicability of the model in prediction of polyphenol and flavonoid responses.

To visualize the relationship between the response and experimental levels of the independent variables for the total phenolics and flavonoid extraction, the response surface plots were constructed according to the quadratic polynomial model equations and reported in Figures S1 and S2.

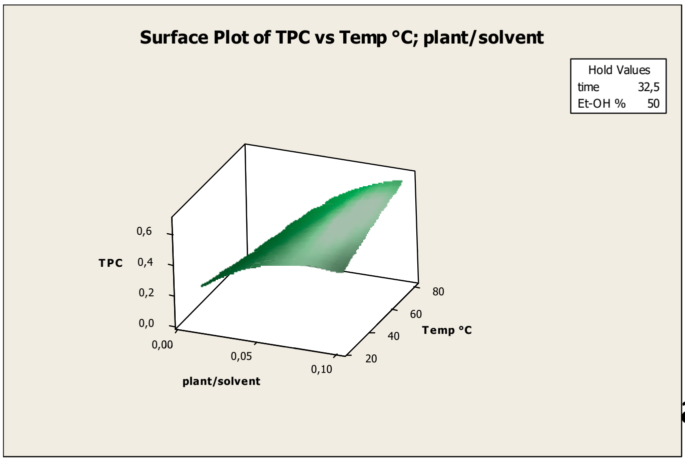
Surface Plot of TPC vs Temp °C; plant/solvent
Hold Values
time 32,5
Et-OH % 50
TPC
0,6
0,4
0,2
0,0
0,00
0,05
0,10
plant/solvent
20
40
60
80
Temp °C

a

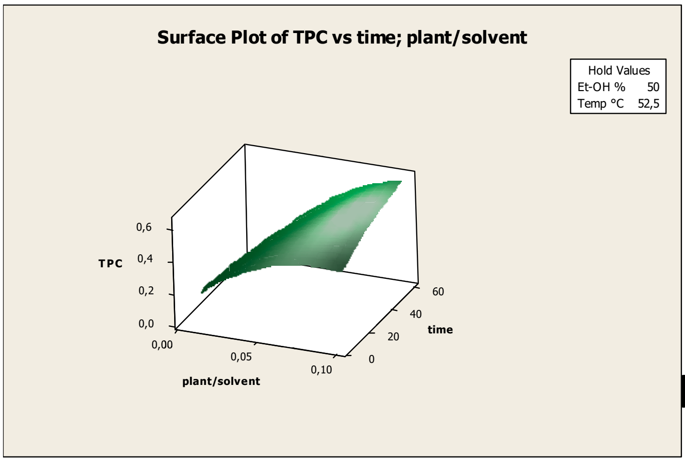
Surface Plot of TPC vs time; plant/solvent
Hold Values
Et-OH % 50
Temp °C 52,5
TPC
0,6
0,4
0,2
0,0
0,00
0,05
0,10
plant/solvent
0
20
40
60
time

b

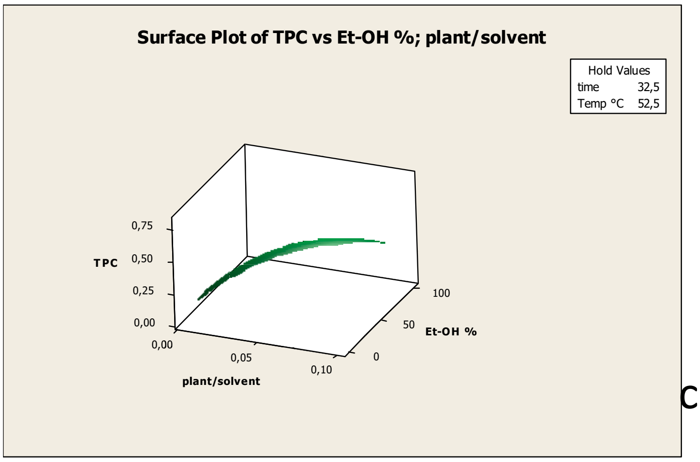
Surface Plot of TPC vs Et-OH %; plant/solvent
Hold Values
time 32,5
Temp °C 52,5
TPC
0,75
0,50
0,25
0,00
0,00
0,05
0,10
plant/solvent
0
50
100
Et-OH %

c

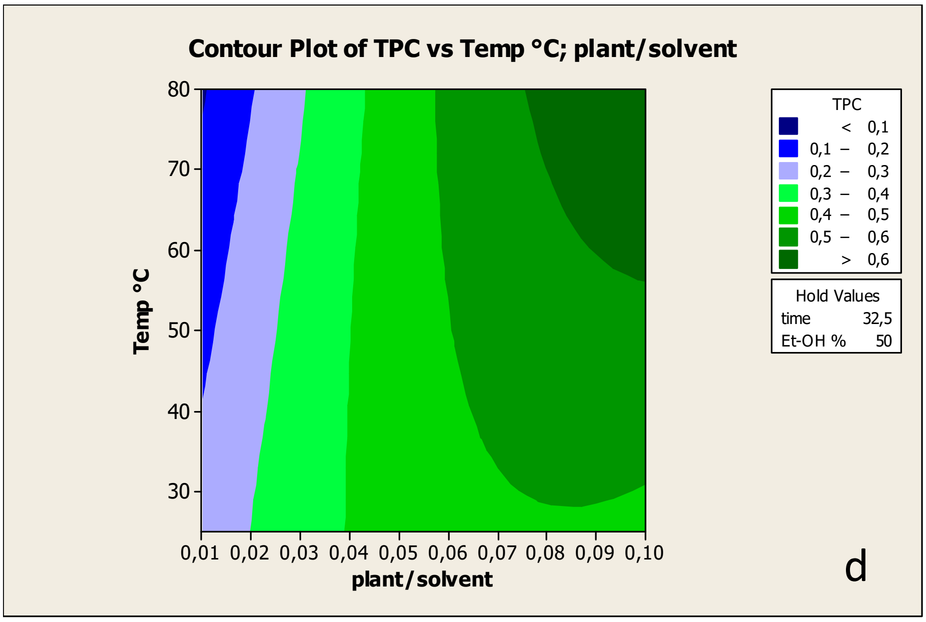

Figure S1. Effects of critical factors on polyphenols extraction: (a-c) Response surface graphs show the effects of the interaction of plant solvent ratio with other variables and (d) Contour graphs of temperature and plant/solvent effect at 32.5 min time and 50% ethanol

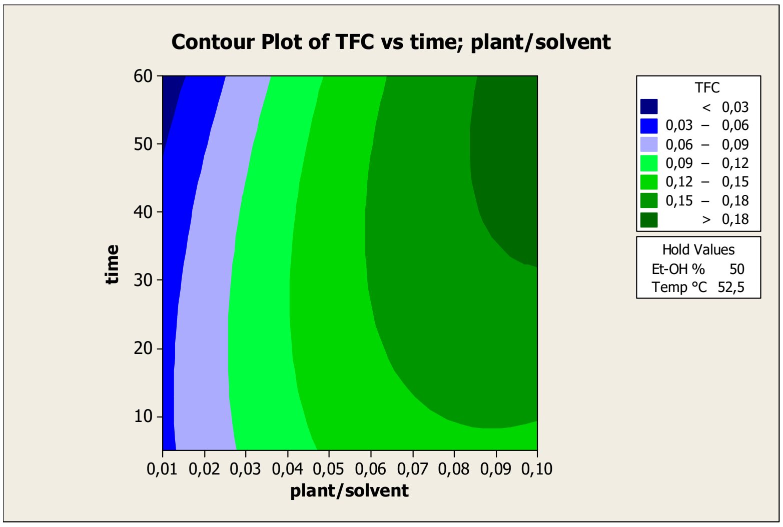

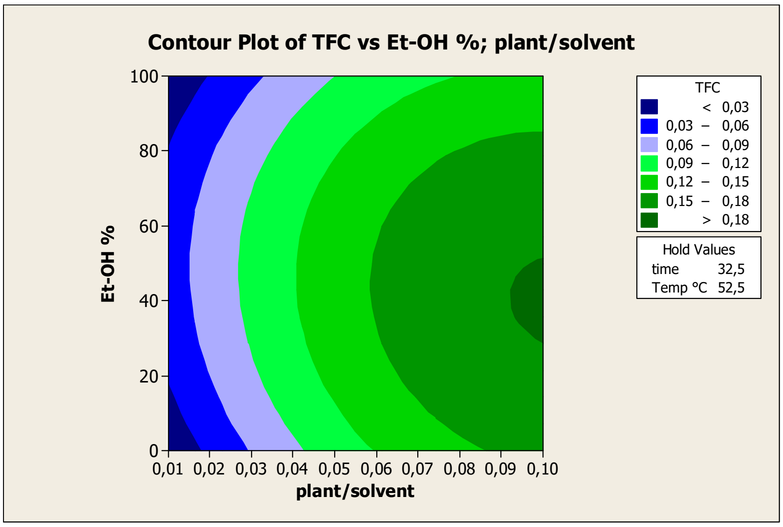

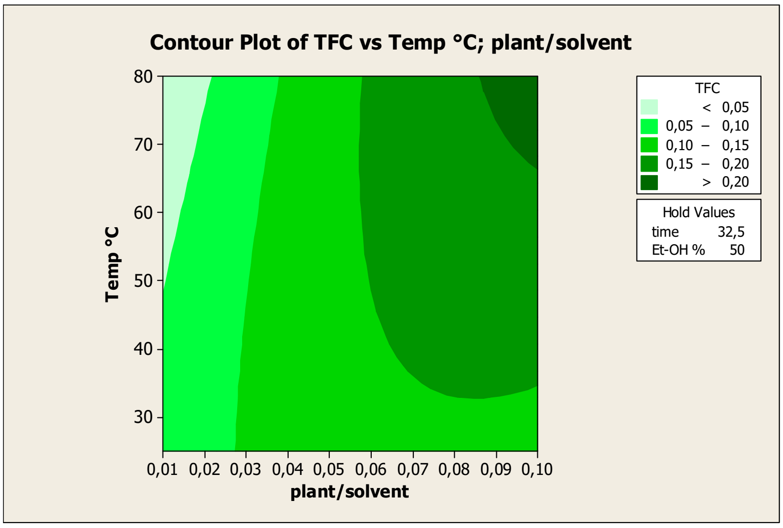

Figure S2. Effect of critical factors on total flavonoids extraction: Contour graphs of plant/solvent interaction with other variables at central point level (32.5 minutes, 50% ethanol, temperature 52,5°C)

The statistical analysis highlighted critical factors, which significantly influence the polyphenols extraction; the factors were plant/solvent ratio, temperature and the interactions of plant/solvent ratio with other variables (plant/solvent, time, temperature, % of ethanol). For the extraction of flavonoids, the significant variables ($p<0,05$) result the plant/solvent ratio alone, the quadratic interaction of plant/solvent and percentage of ethanol (plant/solvent *plant/solvent and Et-OH%*Et-OH%) and as well as the interaction of the factor plant/solvent with time or temperature.

The critical factors should be selected for the optimization of the extraction process considering that the positive or negative effects of selected variables in the mass transfer is not directly related to the physical and chemical characteristics of the solvent, saturation effects or the chemical structure of metabolites present in plant material. In each extraction system, a complex interaction between raw material and solvent system reflects different behavior, which cannot be predicted (Pinelo et al., 2005). The predictive extraction method for total phenols and total flavonoids was validated through a test for response optimizer, setting the software for the optimal desirability and with the weights of responses all 1.0. The variables interactions were tested to an arbitrary target consisting in mean values detected for the phenolics and flavonoid secondary metabolites. In Table 2 are reported the optimal plant/solvent ratio, duration of extraction, percentage of ethanol in the hydroalcholic solution and operative temperature for targeting the responses. They were 1 g of plant in 20 mL of ethanol, extracted in sonicator bath for 5 min at 25°C.

The corresponding predicted response values for phenolics and flavonoids were 0,075 mg of gallic acid equivalents/g and 0,044 rutin equivalents mg/g, respectively. Experiments were run at the recommended conditions and results were expressed as means ± standard deviation. The values obtained experimentally for both response variables resulted not statistically different from the predicted values; thus confirming the validity of the experimental model for polyphenols and flavonoids extraction (Table S2).

Table S2. validation of the experimental model with expected responses and the experimental data.

Operative parameters	Target response	Experimental response (mg/mL, mean±SD)

**PLANT/SOLVENT**	1g/50mL	TPC 0.075 mg/mL	0.069±0.008
TIME	5 min	TFC 0.040 mg/mL	0.036±0.009
Et-OH %	100 %		
TEMPERATURE	25°C		

The liquer Mirinello

In order to test the experimental extraction, a comparison with liqueur samples in terms of phytochemical profile was performed.

Commercial samples from different producers, namely Mirinello Liquori Srl, Carlucci Food and antichi sapori federiciani were bought in a local market in Torremaggiore (Apulia, Italy). A fourth sample was a homemade Mirinello non intended for commercial use. For all samples, the original recipe is unknown. An aliquot of each commercial sample was transferred in anonymous glass bottle and randomly assigned to a number.

In Table S3 is reported the results of phytochemical analysis of liquers.

Table S3. chemical profile of Mirinello liqueur samples. Data are mean value ±SD of triplicate measure.

**Liqueur**	**TPC (mg GAE/mL)**	**TFC (mg rutin/mL)**	**TTC (mg tannic aid/mL)**
HOME MADE	0,17±0.004	0,04±0.008	0,15±0.008
1	0,20±0.002	0,06±0.007	0,19±0.008
2	0,09±0.003	0,02±0.004	0,09±0.005
3	0,07±0.001	0,001±0.001	0,08±0.006

Results highlight a quantitative difference between the home-made and sample 1 compared to the others which are characterized by significant lower amount of all class of secondary metabolites tested. The discriminant between the two set of samples resulted also evident by direct recording of the UV-Vis spectra of liqueur samples (Figure S3a). The spectra of water, ethanol 20%, ethanol 50% and ethanol 100% extracts, as reported in Figure S3b, confirm relevant effects on phytochemical extraction related to the solvent; thus confirming the positive effects of the presence of water in the solvent of extraction to obtain a preparation qualitative and quantitative close to the profile of homemade liqueur.

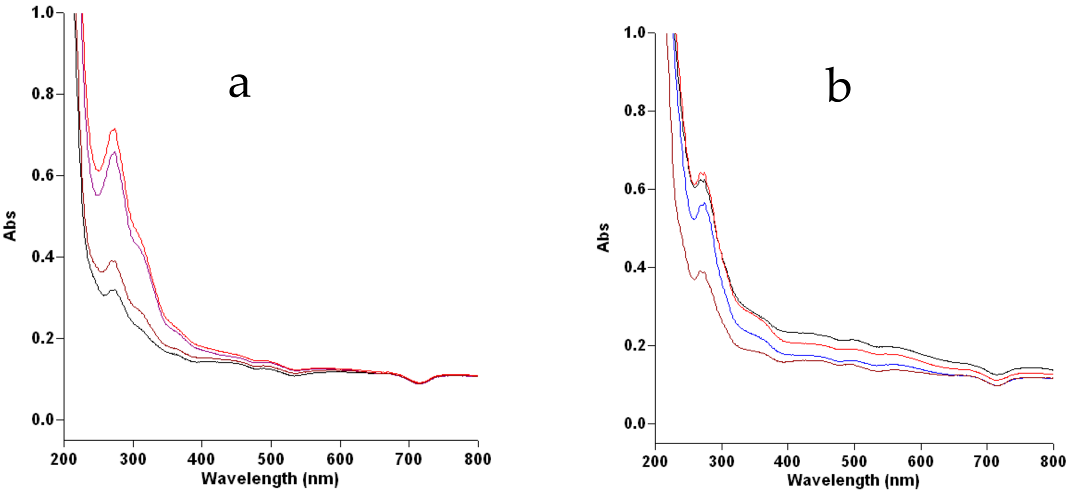

Figure S3. Dilution of liqueurs (**a**), namely home made (purple line) and commercial liqueurs (sample 1- red line, sample 2-black line, sample 3-brown line) and extracts (**b**) from P. mahaleb, 1 mg/mL (water extract, black line; EtOH 20%, blue line; EtOH 50%- red line; EtOH 100%, brown line).

Finally, the response surface model was temptative applied for an *ex-post* interpretation of the recipe used for liqueur preparation. The software response optimizer function was used for a predictive extraction where the expected target represents the experimental values of total polyphenol content and total flavonoid content detected in the liqueur samples.

SAMPLE		homemade	Liqueur 1	Liqueur 2	Liqueur 3
Predicted response	TPC	0.170	0.20	0.09	0.07
	TFC	0.040	0.06	0.02	0.01
Global Solution	plant/solvent	0,0139	0,035	0,014	0,019
	Time (min)	5	60	56.847	60
	Et-OH %	100	85.21	74.95	76.339
	Temp °C	30.813	80	51,111	80
Composite desiderability		1.000	1.000	0.997	1.000

The calculated conditions to obtain an extract similar, in terms of TPC and TFC, to the tested samples do not highlight similarities between the extraction condition for the homemade and sample 1 rather that samples 3 and 4. Conversely, parameters such as cold temperature appear rationale with low temperature of extraction for the homemade product, which is usually performed at room temperature. For all samples, it is required a high percentage or exclusive use of ethanol for extraction resulting coherent with the antifermentative effects of the solvent itself. For homemade preparations, the time is not a relevant parameter and extractions are performed for weeks or somethime months. In this case of the homemade target, the time results really short and surely not coherent with practical preparation. The speculative approach tested did not permit to highlight a clear relationship between phytochemical target and operative condition resuting as a non efficient method to unravel the secret of the liqueur recipe. Furthermore, the interpretation of traditional products only as a merely phytochemical combination should result reductive because a plethora of other factors can influence the chemical composition and the more relevant parameters that for food products are the organoleptic properties.

Reference

Pinelo M., Rubilar M., Jerez M., Sineiro J., Nunez M.J. Effect of solvent, temperature, and solvent-to-solid ratio on the total phenolic content and antiradical activity of extracts from different components of grape pomace. J. Agric. Food Chem. 2005;53:2111–2117. doi: 10.1021/jf0488110.)
